# Perfluoroalkyl acids and time to pregnancy revisited: An update from the Danish National Birth Cohort

**DOI:** 10.1186/s12940-015-0040-9

**Published:** 2015-07-07

**Authors:** Cathrine Carlsen Bach, Zeyan Liew, Bodil Hammer Bech, Ellen Aagaard Nohr, Chunyuan Fei, Eva Cecilie Bonefeld-Jorgensen, Tine Brink Henriksen, Jørn Olsen

**Affiliations:** Perinatal Epidemiology Research Unit, Aarhus University Hospital, Skejby, Denmark; Department of Epidemiology, Fielding School of Public Health, University of California, Los Angeles, USA; Section for Epidemiology, Department of Public Health, Aarhus University, Aarhus, Denmark; Institute of Clinical Research, University of Southern Denmark, Odense, Denmark; Department of Global Surveillance & Pharmacoepidemiology, AbbVie Inc, North Chicago, USA; Centre for Arctic Health & Unit for Cellular and Molecular Toxicology, Department of Public Health, Aarhus University, Aarhus, Denmark; Department of Paediatrics, Aarhus University Hospital, Skejby, Denmark

**Keywords:** Female infertility, Fecundity, Reproduction, Perfluorooctane sulfonate, Perfluorooctanoate, Perfluorinated chemicals, Epidemiology, Humans

## Abstract

**Background:**

We previously demonstrated an association between plasma perfluorooctane sulfonate (PFOS) and perfluorooctanoate (PFOA) and longer time to pregnancy (TTP) in a sample from the Danish National Birth Cohort (DNBC, 1996-2002). In this study we investigated this association in a new sample from the same cohort.

**Methods:**

Sample 1 consisted of 440 women, and Sample 2 consisted of 1161 women from whom we previously published the associations between PFOS or PFOA and TTP. We performed sample-specific and pooled analyses using discrete-time survival analyses to estimate fecundability ratios according to PFOS and PFOA quartiles, adjusted for potential confounders chosen guided by a directed acyclic graph. We also estimated odds ratios for infertility (TTP > 12 months or infertility treatment) according to PFOS and PFOA by multivariable logistic regression.

**Results:**

In Sample 1 PFOS was not associated with lower fecundability ratios or infertility, and there was a tendency towards longer TTP with increasing PFOA only in parous women. In Sample 2 previously reported associations were again seen. In the pooled analyses including both parous and nulliparous women fecundability ratios were 13-22 % lower for the three higher quartiles of PFOS or PFOA compared to the reference quartile.

**Conclusions:**

The pooled analyses were driven by the larger old sample, but we did not corroborate our previous finding of an association between high PFOS and longer TTP in the new sample. The tendency towards an association for PFOA and TTP in parous women may be due to reverse causation. Results from the new sample are more in line with the recent literature.

**Electronic supplementary material:**

The online version of this article (doi:10.1186/s12940-015-0040-9) contains supplementary material, which is available to authorized users.

## Background

Fertility impairment is common and has severe consequences for the affected individuals. Approximately 10 % of all couples experience infertility lasting more than 12 months [1]. Several environmental and lifestyle factors influence human fertility, and perfluoroalkyl acids (PFAAs) may be among these. PFAAs are a group of persistent environmental chemicals that have been used in a wide range of products such as textiles including clothing and carpets, footwear, non-stick pots and pans, and food packaging since the 1950s [2]. Even though the production of specific PFAAs [perfluorooctane sulfonate (PFOS) and perfluorooctanoate (PFOA)] has ceased in parts of the world [3–7], they are resistant to degradation in the environment and accumulate in the human organism [8]. Furthermore, these compounds still occur in some imported products [9], and they are being replaced by other compounds with similar chemical structures.

A few studies have investigated the association between exposure to PFAAs and fecundability measured by the time to pregnancy (TTP) in women [10–14]. TTP is defined as the number of months, or menstrual cycles, it takes for a sexually active couple to conceive from discontinuance of contraception. In our previous study from the Danish National Birth Cohort (DNBC) we found a strong association between exposure to PFOS or PFOA and longer TTP [11]. Specifically, fecundability odds ratios (FORs) were decreased by 30-40 % for the three higher PFOS and PFOA quartiles compared to the lowest. However, subsequent studies reported conflicting results [10, 12–14]. Some studies found an association between PFAAs and TTP in parous, but not in nulliparous women, and potential reverse causality in parous women was discussed [12, 13, 15, 16]. In this study, we examined if our previous findings on the associations between exposure to PFOA and PFOS and TTP could be replicated in an independent new sample of women from the DNBC. We also reanalysed the data from our previous study using a statistical strategy guided by directed acyclic graphs (DAGs). In addition, we conducted pooled analyses of the two samples providing the largest sample size to date in population-based studies of the associations between PFAAs and TTP.

## Methods

### Setting

The DNBC is a nationwide cohort study that included more than 100,000 pregnancies from 1996 to 2002; the details have been described previously [17]. Approximately half of all general practitioners in Denmark took part and recruited pregnant women during early pregnancy. About 60 % of the invited women participated. They donated blood samples during the first and second trimesters and provided information through structured telephone interviews twice during pregnancy and twice after birth. The questionnaires used for the four interviews are available at www.dnbc.dk.

### Participants

**Fig 1:**
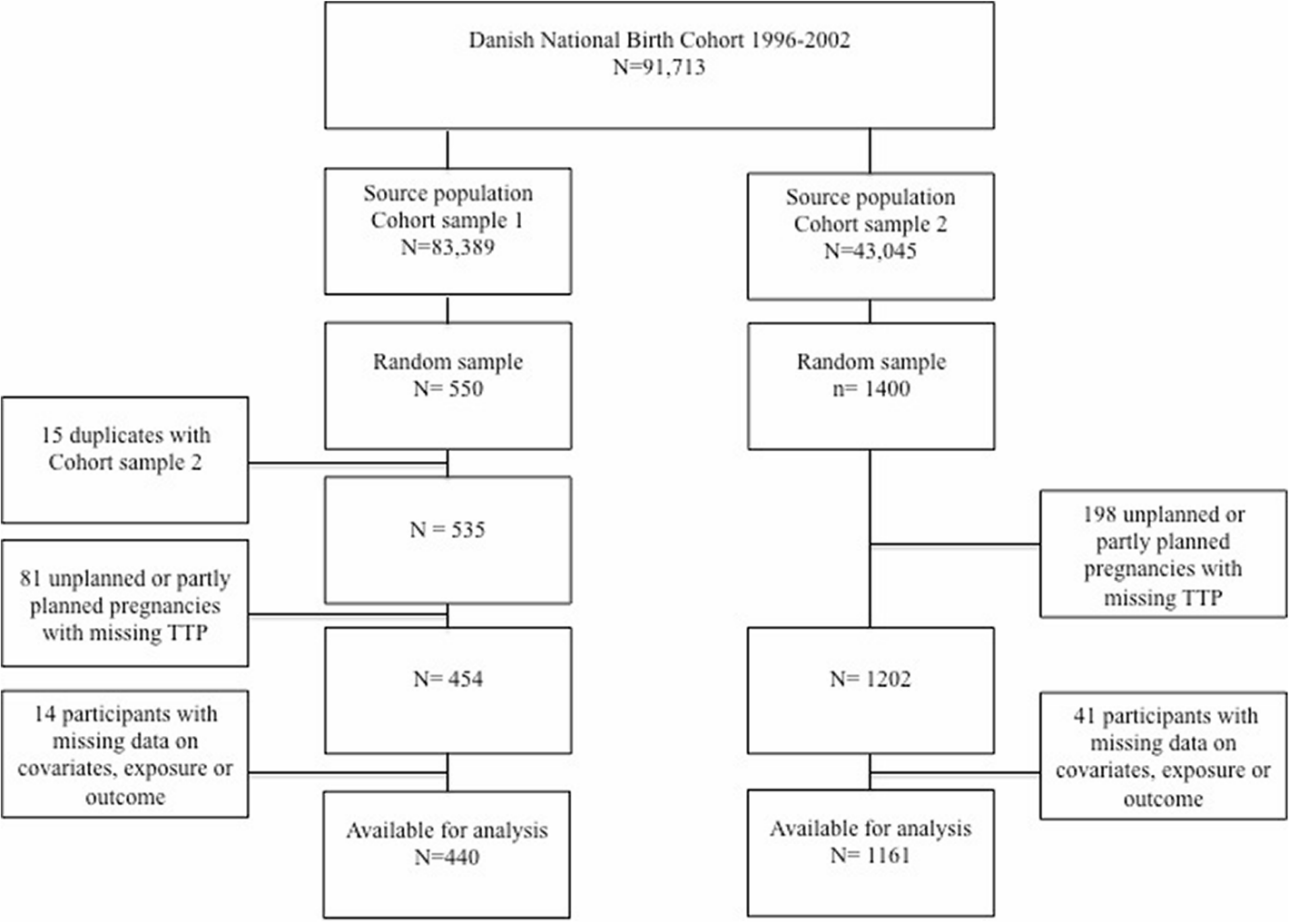
Flowchart of participants in two participant samples from the Danish National Birth Cohort

We studied two samples that were selected independently from the DNBC during 1996-2002 (Fig. 1). The source population for Sample 1 was women who gave birth to a live born singleton, participated in the first telephone interview, and provided a blood sample in the first or second trimester (*n* = 83,389). From these we randomly selected 550 participants who also served as controls in a case-cohort study [18]. Women who gave birth to boys were oversampled since the studied outcomes of the case-cohort study have unequal sex ratios (offspring sex is unlikely to be related to the investigated association).

Sample 2 (*n* = 1400) was randomly selected amongst participants who provided the first blood sample during pregnancy, gave birth to a live born singleton without congenital malformations, and completed the four scheduled interviews (*n* = 43,045). In this sample we previously reported associations between PFOS and PFOA and time to pregnancy [11].

We excluded 81 women from Sample 1 and 198 women from Sample 2 since they did not plan their pregnancy, or reported partly planning but did not provide a TTP. Participants with missing values for exposure, outcome (planned pregnancy without a reported TTP), or covariates (3 % in total) were further excluded from the main analysis. Finally, 440 participants were available for analysis in Sample 1, and 1161 participants in Sample 2 (Fig. 1).

### Exposure assessment

Blood samples from the women were collected by their general practitioners during pregnancy and sent by mail at ambient temperature to Statens Serum Institute. The duration of transport was four to 48 h, but most samples arrived within 28 h. Upon arrival, samples were stored in freezers at -20 °C, -80 °C, or in liquid nitrogen. Plasma from Sample 1 was analysed in 2013 at the Department of Environmental Science, Aarhus University, Denmark. In Sample 2 we measured plasma concentrations of PFOS and PFOA at the 3M Toxicology Laboratory in 2007 [11]. The details of the laboratory procedures and equipment were described in a previous paper [19]. Both laboratories were blinded to participant information and used similar analytical techniques. PFAAs were abstracted from plasma by solid phase extraction, and the concentrations of PFAAs were assessed by liquid chromatography–tandem mass spectrometry. For Sample 1, one blood sample was missing and could therefore not be analysed for PFAA content. For Sample 2, the analytic results for five samples were not usable and were thus excluded. Fifteen samples overlapped between the two participant samples and were analysed in both laboratories for comparison. In the statistical analyses the women who donated these samples were included in Sample 2. Measured concentrations in the duplicate samples were on average 9.6 ng/mL lower for PFOS and 1.4 ng/mL lower for PFOA at Aarhus University compared to the 3M laboratory [18]. Though the concentrations measured at the two laboratories differed they were highly correlated [Pearson correlation coefficient r = 0.94 for PFOS and r = 0.95 for PFOA [18]]. All values were above the limit of quantification (LOQ) in Sample 1, and for Sample 2, all values were above the LOQ, except one PFOA value that was assigned half the LOQ.

### Outcomes

TTP was reported in the first interview performed at approximately 15 weeks of gestation. Women were asked whether their pregnancy was planned, partly planned or not planned. The women who answered “planned” or “partly planned” were asked how long they tried to get pregnant before they succeeded, and five response categories were provided: pregnant immediately (<1 month), after 1-2, 3-5, 6-12, or after > 12 months. We defined infertility as a TTP above 12 months or infertility treatment for the current pregnancy.

### Statistical analyses

#### Main analyses

Analyses on PFOA and PFOS and the TTP or infertility were performed by each sample separately and as pooled analyses combining the two samples. We performed all analyses with and without stratification by parity (nulliparous versus parous women). Concentrations of PFOS and PFOA were divided into quartiles, and we used the lowest quartile as reference. To make the numbers in each quartile evenly distributed, and to take different measurements between the two laboratories into account, we generated quartiles for each participant sample, including parity-specific quartiles for the parity-stratified analyses. In the 15 duplicate samples from the two laboratories women would generally end up in the same quartile. All analyses were also performed using natural log-transformed continuous exposure concentrations.

To estimate fecundability ratios, we used a discrete-time survival model with a complementary log-log link. The fecundability ratio (FR) is the probability of conceiving in a given interval in a group with higher exposure compared to the reference group, conditionally on not having conceived in the previous period. Thus, a FR below one indicates impaired fecundability as measured by a longer TTP. Women who received infertility treatment were added to the highest TTP category. We also performed multivariate logistic regression analyses to estimate infertility odds ratios.

Selection of covariates was based on a DAG (Additional file 1 Fig. 1) and included age (continuous), pre-pregnancy body mass index (BMI, continuous), socio-occupational status (higher versus middle/lower), and parity (primiparous or multiparous). For the pooled analyses of the two samples we additionally adjusted for the sample (by use of a dummy variable assigning each of the two samples with a different value) in order to account for differences in sampling and laboratories. The data source for maternal age was the Danish Medical Birth Registry, and for pre-pregnancy BMI and parity, data was obtained from the first questionnaire in the DNBC. Socio-occupational status was grouped based on maternal education and job reported in the cohort or, if this information was missing, the corresponding paternal information. The statistical analyses were conducted using STATA statistical software version 13 (StataCorp, College Station, TX, USA).

#### Sensitivity and bias analyses

Couples that had unplanned pregnancies, or pregnancies with missing TTP for other reasons, may have either high or low biological fecundity. We therefore performed bias analyses that included these pregnancies without a valid TTP in the lowest as well as in the highest TTP categories in order to examine the impact of excluding women with missing TTP from our study population.

We also restricted our analyses to women that provided a blood sample before 14 weeks of gestation since maternal plasma concentrations of PFAAs decrease during pregnancy, and therefore exposure assessment may become less comparable with increasing pregnancy duration. In order to take potential bias by the unequal offspring sex ratio into account in Sample 1, we used inverse probability weighting. Whether to condition on parity is a controversial issue, and in order to compare our results with other studies, we also showed estimates adjusted for age, BMI and socio-occupational status, but not parity.

We did a posthoc analysis in Sample 1 restricted to women who participated in all four interviews to assess whether this restriction in Sample 2 could have caused selection bias.

### Ethical approval

The study was approved by the Danish Data protection Agency (references 2012-41-1288 and 2006-41-6324), and the Danish National Committee on Health Research Ethics (reference M-20110054).

## Results

Characteristics of study participants are listed in Table 1. Maternal age, parity, BMI, and socio-economic status as well as the year of inclusion were comparable between the two samples. A slightly higher proportion of women had a TTP above 12 months or received infertility treatment for the current pregnancy in Sample 2. Measured average PFOS and PFOA levels were slightly lower for Sample 1. Quartile limits for PFOS and PFOA are listed by sample in Additional file 1 Table 1.

**Table 1:** Participant characteristics for women from two samples of the Danish National Birth Cohort

	Sample 1 (n = 440)			Sample 2 (n = 1161)		
	Percentage	Median	Interquartile range	Percentage	Median	Interquartile range
PFOS (ng/mL)		27.9	21.0 - 36.2		34.2	26.9 - 43.8
PFOA (ng/mL)		4.0	3.0 - 5.5		5.4	4.0 - 7.1
Age at delivery (years)		29	27 - 33		30	27 - 33
Parity	0	1+				
	48			45		
	52			55		
Pre-pregnancy BMI (kg/m^2^)		22	21 - 25		23	21 - 26
SES	Higher	Middle/lower				
	50			52		
	50			48		
Time to pregnancy (months)	<1	1-2	3-5	6-12	>12	
	26			23		
	24			25		
	22			20		
	15			16		
	13			16		
Infertility	13			17		

Abbreviations: Perfluorooctanoate (PFOA), perfluorooctane sulfonate (PFOS), Body Mass Index (BMI), socio-economic status (SES)

Medians are listed for continuous variables and percentages for categorical variables. Infertility was defined as a time to pregnancy >12 months or receiving infertility treatment

SES definitions: Higher SES includes women with managerial posts and/or a job requiring a long or medium-cycle higher education. Middle/lower SES includes women who have a job requiring a shorter education, who do not have an education, or who do not have a job

Missing values: One for age in Sample 2, none in Sample 1. None for parity in Sample 2 and one in Sample 1. For BMI 33 values were missing for Sample 2, and six values were missing for Sample 1. For SES, four values were missing for Sample 2, and two for Sample 1. Nine women from Sample 2 and three women from Sample 1 with planned pregnancies had missing time to pregnancy. One woman from Sample 2 had missing data on both planner status and time to pregnancy

### Perfluoroctane sulfonate and time to pregnancy and infertility

In the pooled analysis for PFOS, fecundability was approximately 15 % lower in the three higher quartiles compared to the reference (Table 2). In Sample 2, fecundability ratios were slightly lower than for the pooled sample, while in Sample 1, PFOS was not associated with lower fecundability. After stratification by parity, associations remained close to null in Sample 1. The estimates were slightly lower among nulliparous women, but were attenuated in parous women from both Sample 2 and the pooled samples, compared with those observed in all women (Table 3).

**Table 2:** Fecundability ratios according to plasma PFOS and PFOA

		Sample 1			Sample 2			Pooled analysis		
		Crude FR	Adjusted^a^ FR	95 % CI	Crude FR	Adjusted^a^ FR	95 % CI	Crude FR	Adjusted^b^ FR	95 % CI
PFOS										
	Q1	1.00	1.00		1.00	1.00		1.00	1.00	
	Q2	1.06	1.08	0.81 - 1.44	0.79	0.79	0.66 - 0.95	0.85	0.87	0.75 - 1.02
	Q3	0.99	0.99	0.73 - 1.34	0.80	0.78	0.65 - 0 .95	0.84	0.85	0.72 - 0.99
	Q4	0.91	0.99	0.74 - 1.33	0.75	0.78	0.65 - 0.94	0.79	0.85	0.72 – 0.99
	Log	0.94	0.96	0.75 – 1.24	0.74	0.76	0.62 – 0.92	0.79	0.83	0.72 – 0.97
PFOA										
	Q1	1.00	1.00		1.00	1.00		1.00	1.00	
	Q2	0.91	0.92	0.69 - 1.22	0.75	0.78	0.65 - 0.94	0.79	0.81	0.70 - 0.95
	Q3	0.90	0.94	0.71 - 1.26	0.80	0.83	0.69 - 1.00	0.82	0.86	0.74 - 1.01
	Q4	0.74	0.86	0.63 - 1.19	0.69	0.74	0.60 - 0.90	0.70	0.78	0.66 - 0.92
	Log	0.75	0.89	0.68 – 1.15	0.69	0.72	0.61 – 0.85	0.70	0.77	0.67 – 0.89

^a^Adjusted for age, socio-economic status, body mass index, and parity. ^b^Additionally adjusted for sample

Abbreviations: Perfluorooctane sulfonate (PFOS), perfluorooctanoate (PFOA), quartile (Q), log-transformed continuous exposure levels (Log), fecundability ratio (FR), 95 % confidence interval (95 % CI)

The fecundability ratio denotes the probability of conceiving in a given interval in a group of women with higher exposure compared to the reference group, conditionally on not having conceived in the previous period

**Table 3:** Fecundability ratios according to plasma PFOS and PFOA by parity

		Sample 1			Sample 2			Pooled analysis		
		Crude FR	Adjusted^a^ FR	95 % CI	Crude FR	Adjusted^a^ FR	95 % CI	Crude FR	Adjusted^b^ FR	95 % CI
PFOS										
N	Q1	1.00	1.00		1.00	1.00		1.00	1.00	
	Q2	1.16	1.16	0.77 - 1.75	0.97	0.89	0.68 - 1.17	1.02	0.98	0.78 - 1.23
	Q3	1.07	1.01	0.65 - 1.57	0.80	0.68	0.52 - 0.91	0.87	0.78	0.62 – 1.00
	Q4	0.98	0.97	0.62 - 1.51	0.79	0.69	0.53 - 0.91	0.84	0.79	0.63 – 0.99
	Log	1.06	1.02	0.72 – 1.44	0.75	0.62	0.47 – 0.83	0.86	0.78	0.63 – 0.97
P	Q1	1.00	1.00		1.00	1.00		1.00	1.00	
	Q2	0.91	1.04	0.69 - 1.55	0.86	0.88	0.69 - 1.12	0.88	0.92	0.75 - 1.14
	Q3	0.95	1.05	0.69 - 1.60	0.71	0.72	0.56 – 0.94	0.77	0.80	0.65 – 0.99
	Q4	0.89	1.04	0.70 - 1.55	0.84	0.90	0.70 - 1.14	0.85	0.93	0.76 - 1.15
	Log	0.82	0.91	0.63 – 1.30	0.79	0.85	0.66 – 1.09	0.77	0.86	0.70 – 1.06
PFOA										
N	Q1	1.00	1.00		1.00	1.00		1.00	1.00	
	Q2	0.88	0.82	0.53 – 1.26	1.09	0.93	0.71 - 1.23	1.03	0.90	0.71 - 1.13
	Q3	1.15	1.11	0.73 – 1.69	0.94	0.80	0.71 - 1.07	0.99	0.90	0.71 - 1.13
	Q4	0.98	0.99	0.64 – 1.54	0.82	0.74	0.56 - 0.98	0.86	0.82	0.65 - 1.04
	Log	1.23	1.26	0.86 – 1.85	0.73	0.67	0.51 – 0.88	0.87	0.84	0.67 – 1.05
P	Q1	1.00	1.00		1.00	1.00		1.00	1.00	
	Q2	1.19	1.30	0.86 – 1.98	0.78	0.76	0.59 - 0.96	0.85	0.86	0.70 – 1.06
	Q3	0.93	0.96	0.66 - 1.41	0.69	0.71	0.56 – 0.90	0.74	0.78	0.63 - 0.94
	Q4	0.70	0.74	0.48 - 1.13	0.76	0.78	0.61 – 0.99	0.74	0.76	0.61 – 0.94
	Log	0.64	0.66	0.46 – 0.95	0.74	0.76	0.61 – 0.93	0.68	0.72	0.61 – 0.87

^a^Adjusted for age, socio-economic status, and body mass index. ^b^Additionally adjusted for sample

Abbreviations: Perfluorooctane sulfonate (PFOS), perfluorooctanoate (PFOA), nulliparous women (N), parous women (P), quartile (Q), log-transformed continuous exposure levels (Log), fecundability ratio (FR), 95 % confidence interval (95 % CI)

### Perfluoroctanoate and time to pregnancy and infertility

For PFOA, results from the pooled analyses were similar to those for PFOS. The associations were stronger in Sample 2 compared to Sample 1. Fecundability ratios did not differ much between nulliparous and parous women in the pooled sample and in Sample 2, but in Sample 1 there was a tendency towards longer time to pregnancy in parous, but not in nulliparous women (Table 3). Infertility odds ratios were generally higher with exposure to higher concentrations of PFOS or PFOA in Sample 2 and the pooled sample, however the confidence intervals were wider than for the fecundability odds ratios. In Sample 1 the association was less clear (Additional file 1 Tables 2 and 3).

### Sensitivity and bias analyses

When we included women with missing TTP in the lowest TTP category, results remained unchanged, but when we added them to the highest TTP category, no clear associations between exposure to PFOS or PFOA and the TTP were evident (Additional file 1 Table 4). Restriction to participants, who provided a blood sample before 14 weeks of gestation [*n* = 403 (92 %) for Sample 1; *n* = 1147 (99 %) for Sample 2], did not change our results. Neither did inverse probability weighting for offspring sex in Sample 1. FRs were slightly lower in analyses not adjusted for parity (Additional file 1 Table 5).

In the posthoc analysis in Sample 1, restricted to women, who participated in all four interviews, we found estimates similar to the main analysis with wider confidence intervals (Additional file 1 Table 6).

## Discussion

The reanalysis of data from our previous study as well as the pooled analyses indicated as before an association between exposure to PFOS or PFOA and TTP, independent of parity. However, results from the new Sample 1 did not support an association between PFOS and TTP while for PFOA, there was a tendency towards an association with TTP in parous women, but not in nulliparous women.

Sample 1 was smaller than Sample 2 and therefore more prone to random fluctuations as illustrated by the wider confidence intervals, but the results from this sample were more similar to results from most previous studies [10, 12–14]. Differences between the two samples may be due to random forces or systematic differences, for instance due to differences in sampling or use of different laboratories. It is notable that women in the lowest PFAA quartile in Sample 2 had very short TTP, and no clear dose-response pattern was seen in the main quartile analyses. Such a pattern was present in Sample 2 among nulliparous women upon stratification.

Results from our reanalysis of Sample 2 were consistent with results from our previous study [11] as expected, even though we used different statistical approaches, including a different set of covariates. Two smaller pregnancy planner studies, Vestergaard et al. (2012) from Denmark and Buck Louis et al. (2013) from the USA, followed couples that attempted to conceive for six and 12 months, respectively. They found no associations between exposure to PFOS or PFOA and fecundability odds ratios or infertility with point estimates very close to 1. Vestergaard et al. (2012) only included nulliparous women while Buck Louis et al. (2013) included parous women as well. Jørgensen et al. (2014) studied women from Greenland, Poland, and Ukraine and found no association between PFOA and fecundability or infertility. In their analyses including both nulliparous and parous women there was a tendency towards lower fecundability and infertility with increasing PFOS, but this tendency disappeared when they restricted to nulliparous women. A Norwegian case-control study by Whitworth et al. (2012) found increased odds for infertility with exposure to PFOS or PFOA only in parous women, which they suggested could reflect reverse causality [13]. During pregnancy, delivery and lactation, PFAA concentrations decrease in maternal blood [20]. After this period, concentrations may slowly increase again, and a longer interval between the birth of one child and a subsequent pregnancy may therefore correlate with higher PFAA concentrations [12, 13, 21], which may induce a spurious association between PFAA concentrations and TTP in parous women. Only our results for Sample 1 regarding PFOA supported this explanation, but parity remains an important factor to control since it is likely to be associated with both individual PFAA levels and the TTP (through determinants of individual fecundability, see Additional file 1 Fig. 1).

Blood samples from our two DNBC samples were analysed in two different laboratories, and although paired measurements from the two laboratories were highly correlated, differing measurement errors may play a role. We addressed potential differences between the two laboratories by categorizing exposure before pooling the two samples and adjusting for the sample.

The causal window of interest may be when a couple initiates their attempt to conceive [10, 12]. We measured exposure in the first or second trimester, and levels at that time probably correlated closely with those at initiation of the attempt to conceive. Although PFAA concentrations decrease during gestation [20, 21], gestational age at blood drawing is not likely to be associated with TTP. Analyses with restriction to samples taken before gestational week 14 produced similar results. Samples were transported for one to two days at ambient temperature and stored for several years before analyses, and in spite of the environmental persistency and long half-lives of PFAAs [22] this may be a problem. However, it is unlikely that any measurement error due to ex vivo degradation is associated with TTP, but random measurement error may bias results, most likely towards the null. We did not measure the glomerular filtration rate, which may affect both PFAA levels and the fecundability.

TTP is a measure of couple fecundability. Exposures in men could potentially affect male fertility.

We did not measure PFAA levels in the partners of our participants, but the association between exposures in women and TTP might potentially reflect a causal association between male exposure and fertility. Exposures in couples are expected to be correlated due to similar lifestyle and home environment. The association between PFAA exposure and male reproductive function has been investigated in several studies, but few studies demonstrated any convincing associations [10, 23–30]. We had no information on other determinants of TTP including family planning patterns such as discontinuance of birth control, frequency and timing of intercourse, and persistency in the attempt to become pregnant. However, we do not believe that these factors are directly associated with PFAA levels.

All women in the study gave birth to a live born child. Couples with unresolved infertility, spontaneous abortions, or stillbirths are therefore not studied. We were unable to include women with missing TTP, who were most likely to have conceived unexpectedly, but we found that their concentrations of both PFOA and PFOS were approximately 10 % lower compared to those included. Exclusion of this particular group could underestimate the association if their TTP was short, and vice versa if it was long. However, inclusion of these women in the lowest TTP category did not strengthen the associations (Additional file 1 Table 4). Adding them to the highest TTP category removed the observed associations, which may be explained by the lower PFAA concentrations in these women. Recall of TTP during pregnancy may be imperfect, but the recall time period was short, and differential misclassification concerning TTP is unlikely since participants were unaware of exposure concentrations.

Bias due to the women’s willingness to participate in the cohort is unlikely since women were unaware of their PFAA levels, even though it is possible that selection depended on other factors associated with both PFAA concentrations and the TTP. The main difference between the source populations for the two samples was that Sample 1 was sampled from women, who completed the first interview independently of their completion of the following three interviews, and Sample 2 was sampled from women that completed all four interviews. Therefore, the source population for Sample 2 was approximately half the size of the source population for Sample 1 (Fig. 1). However, baseline characteristics and TTP distributions were similar for women in the two DNBC source populations, who participated in all four interviews (*n* = 43,045) or the first interview independently of participation in the three latter (*n* = 83,389). Both samples seem to be representative for their source populations, even though we observed slightly shorter average TTP in Sample 1 compared with Sample 2. Estimates from Sample 1 restricted to women, who participated in all four interviews, were similar to the main analysis, and therefore we assume that any selection bias in either sample is unlikely to be due to the different sampling strategies.

## Conclusions

We did not corroborate our previous finding of an association between higher PFOS and longer TTP using a new participant sample from the DNBC. Regarding PFOA, we identified an association with TTP only in parous women in this sample. Overall, pooled analyses of the two samples still indicated that high levels of maternal PFOA and PFOS were associated with longer TTP, regardless of parity, but these results were primarily driven by the larger sample from our previous study. Results from the new Sample 1 add uncertainty to the results from our older Sample 2, especially for PFOS. We found no dose-response pattern in Sample 2 in which the associations were mainly based upon short TTP in the low exposure group. Results from the new sample are more in line with the rest of the existing literature, and therefore convincing evidence for an association between exposure to PFOA or PFOS and TTP is still lacking.

## Additional file

Additional file 1: Figure 1. Causal directed acyclic graph on the association between perfluoroalkyl acid exposure and time to pregnancy. **Table 1.** Quartile definitions for PFOS and PFOA in the two samples from the Danish National Birth Cohort. **Table 2.** Infertility odds ratios according to plasma PFOS and PFOA in the Danish National Birth Cohort. **Table 3.** Infertility odds ratios according to plasma PFOS and PFOA by parity in the Danish National Birth Cohort. **Table 4.** Bias analysis of fecundability ratios for PFOS and PFOA including women with missing time to pregnancy in the lowest and highest time to pregnancy group. **Table 5.** Fecundability ratios according to plasma PFOS and PFOA without adjustment for parity. **Table 6.** Fecundability ratios for PFOS and PFOA in Sample 1, unrestricted and restricted to those completing all four interviews.
