# Supplementary material for: Perfluoroalkyl acids and time to pregnancy revisited: An update from the Danish National Birth Cohort
Source: Environ Health. 2015 Jul 7;14:59. doi: 10.1186/s12940-015-0040-9 (PMC4493954; doi:10.1186/s12940-015-0040-9)
Supplement: Additional file 1: Figure 1. — Causal directed acyclic graph on the association between perfluoroalkyl acid exposure and time to pregnancy. Table 1. Quartile definitions for PFOS and PFOA in the two samples from the Danish National Birth Cohort. Table 2. Infertility odds ratios according to plasma PFOS and PFOA in the Danish National Birth Cohort. Table 3. Infertility odds ratios according to plasma PFOS and PFOA by parity in the Danish National Birth Cohort. Table 4. Bias analysis of fecundability ratios for PFOS and PFOA including women with missing time to pregnancy in the lowest and highest time to pregnancy group. Table 5. Fecundability ratios according to plasma PFOS and PFOA without adjustment for parity. Table 6. Fecundability ratios for PFOS and PFOA in Sample 1, unrestricted and restricted to those completing all four interviews. [file 12940_2015_40_MOESM1_ESM.pdf]

Additional File

Additional Figure 1. Causal directed acyclic graph on the association between perfluoroalkyl acid exposure and time to pregnancy.

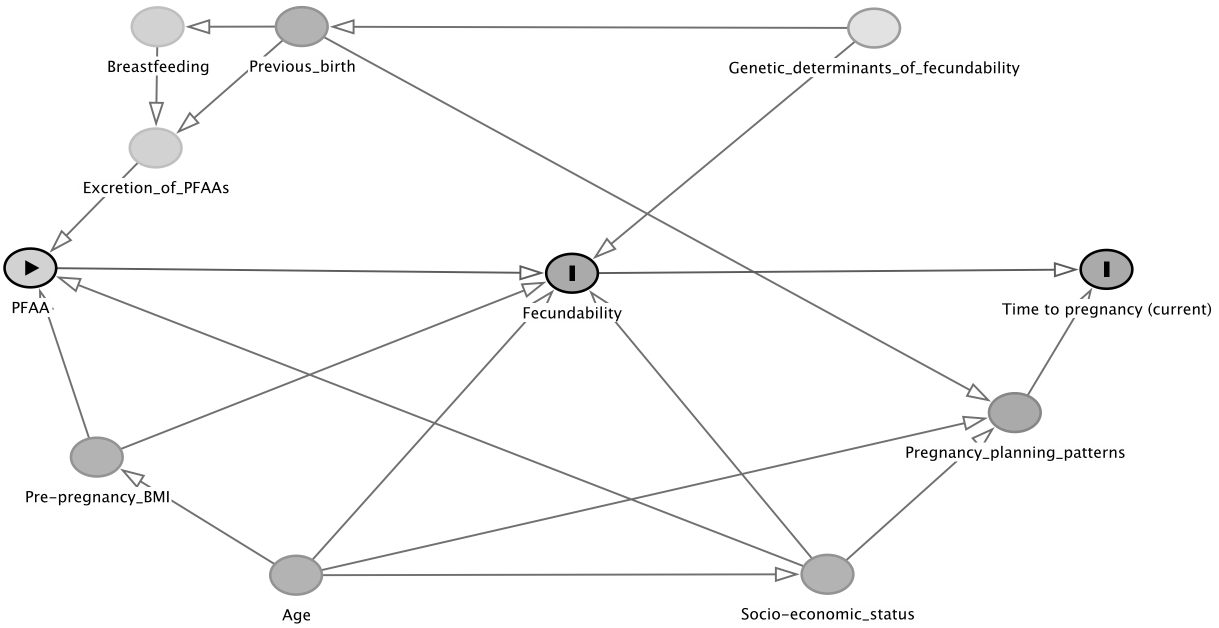

Figure made by use of www.daggity.net (Textor *et al.*, 2011).

Abbreviations: Body mass index (BMI), perfluorooctane sulfonate (PFOS), perfluorooctanoate (PFOA).

Additional Table 1. Quartile definitions for PFOS and PFOA in the two samples from the Danish National Birth Cohort.

	**PFOS**		**PFOA**	
**Quartile**	Sample 1	Sample 2	Sample 1	Sample 2
1	5.6 – 21.0	6.4 – 26.9	0.6 – 3.0	0.5 – 4.0
2	21.1 – 27.8	27.0 – 34.2	3.1 – 4.0	4.1 – 5.4
3	27.9 – 36.2	34.3 – 43.8	4.1 – 5.5	5.4 – 7.1
4	36.3 - 103.8	43.9 – 106.7	5.6 – 17.7	7.2 – 41.5

Abbreviations: Perfluorooctane sulfonate (PFOS), perfluorooctanoate (PFOA).

Additional Table 2. Infertility odds ratios according to plasma PFOS and PFOA in the Danish National Birth Cohort.

		**Sample 1**				**Sample 2**				**Pooled analysis**		
		Infertile (n)	Crude OR	Adjusted[a] OR	95 % CI	Infertile (n)	Crude OR	Adjusted[a] OR	95 % CI	Crude OR	Adjusted[b] OR	95 % CI
**PFOS**	Q1	15	1.00	1.00		35	1.00	1.00		1.00	1.00	
	Q2	11	0.70	0.74	0.31 - 1.75	52	1.60	1.65	1.01 - 2.68	1.32	1.34	0.88 – 2.04
	Q3	15	0.98	1.01	0.45 - 2.28	55	1.75	1.85	1.13 - 3.02	1.50	1.55	1.02 - 2.36
	Q4	16	1.10	1.03	0.46 - 2.33	57	1.80	1.89	1.16 - 3.08	1.59	1.58	1.05 - 2.40
	Log	57	1.03	1.04	0.54 – 2.00	199	1.90	2.04	1.29 – 3.24	1.67	1.75	1.21 – 2.53
**PFOA**	Q1	11	1.00	1.00		31	1.00	1.00		1.00	1.00	
	Q2	13	1.26	1.30	0.53 – 3.19	58	2.12	1.91	1.16 - 3.13	1.88	1.73	1.12 - 2.65
	Q3	12	1.15	1.03	0.41 – 2.59	47	1.64	1.43	0.85 - 2.40	1.51	1.30	0.83- 2.04
	Q4	21	2.21	1.67	0.70 – 4.00	63	2.35	2.07	1.24 – 3.48	2.31	1.94	1.25 - 3.03
	Log	57	1.71	1.18	0.58 – 2.39	199	2.09	1.91	1.25 – 2.91	2.08	2.41	1.72 – 3.38

[a]Adjusted for age, socio-economic status, BMI, and parity. [b]Additionally adjusted for sample.

Abbreviations: Perfluorooctane sulfonate (PFOS), perfluorooctanoate (PFOA), quartile (Q), log-transformed continuous exposure levels (Log), odds ratio (OR), 95 % confidence interval (95 % CI).
Infertility was defined as a TTP above 12 months or infertility treatment for the current pregnancy.

Additional Table 3. Infertility odds ratios according to plasma PFOS and PFOA by parity in the Danish National Birth Cohort.

		Sample 1				Sample 2				Pooled analysis		
		Infertile (n)	Crude OR	Adjusted[a] OR	95 % CI	Infertile (n)	Crude OR	Adjusted[a] OR	95 % CI	Crude OR	Adjusted[b] OR	95 % CI
**PFOS**												
**N**	Q1	11	1.00	1.00		22	1.00	1.00		1.00	1.00	
	Q2	7	0.57	0.64	0.22 – 1.87	24	1.17	1.47	0.74 – 2.91	0.96	1.15	0.65 – 2.04
	Q3	10	0.91	1.13	0.41 – 3.10	33	1.70	2.71	1.38 – 5.30	1.42	2.03	1.17 – 3.53
	Q4	11	1.02	1.23	0.45 – 3.39	29	1.46	2.11	1.08 – 4.15	1.31	1.76	1.01 – 3.07
	Log		0.89	1.15	0.52 – 2.54	108	1.66	2.61	1.32 – 5.16	1.34	1.83	1.10 – 3.04
**P**	Q1	4	1.00	1.00		15	1.00	1.00		1.00	1.00	
	Q2	5	1.20	1.02	0.25 - 4.10	21	1.50	1.44	0.79 – 2.99	1.43	1.33	0.70 – 2.54
	Q3	5	1.25	0.88	0.21 - 3.73	32	2.49	2.44	1.23 - 4.85	2.18	2.03	1.10 – 3.75
	Q4	4	1.00	0.70	0.16 - 3.11	23	1.71	1.60	0.78 – 3.28	1.54	1.37	0.72 – 2.60
	Log		1.13	0.84	0.28 – 2.47	91	1.85	1.77	0.93 – 3.34	1.87	1.51	0.86 – 2.65
**PFOA**												
**N**	Q1	9	1.00	1.00		28	1.00	1.00		1.00	1.00	
	Q2	11	1.34	1.92	0.67 – 5.49	19	0.65	0.75	0.38 – 1.46	0.81	0.97	0.56 – 1.70
	Q3	8	0.89	1.22	0.41 – 3.67	29	1.05	1.39	0.75 – 2.61	1.01	1.32	0.77 – 2.27
	Q4	11	1.34	1.56	0.55 – 4.42	32	1.24	1.48	0.80 – 2.75	1.26	1.46	0.86 – 2.47
	Log		0.77	0.89	0.36 – 2.18	108	1.52	1.77	0.95 – 3.28	1.26	1.39	0.84 – 2.29
**P**	Q1	4	1.00	1.00		12	1.00	1.00		1.00	1.00	
	Q2	2	0.55	0.39	0.06 – 2.40	24	2.23	2.30	1.09 – 4.87	1.80	1.71	0.87 – 3.33
	Q3	4	1.06	0.80	0.18 – 3.52	29	2.75	2.44	1.17 – 5.07	2.30	1.99	1.04 – 3.80
	Q4	8	2.43	1.74	0.46 – 6.55	26	2.45	2.16	1.02 – 4.56	2.45	2.09	1.09 – 3.99
	Log		2.68	2.12	0.68 – 6.64	91	2.27	2.04	1.14 – 3.64	2.62	2.06	1.22 – 3.49

[a]Adjusted for age, socio-economic status, and body mass index. [b]Additionally adjusted for sample.

Abbreviations: Perfluorooctane sulfonate (PFOS), perfluorooctanoate (PFOA), nulliparous (N), parous (P), quartile (Q), log-transformed continuous exposure (Log), odds ratio (OR), 95 % confidence interval (95 % CI).

Additional Table 4. Bias analysis of fecundability ratios for PFOS and PFOA including women with missing time to pregnancy in the lowest and highest time to pregnancy group.

	**Missing in lowest TTP category**						**Missing in highest TTP category**					
	Sample 1		Sample 2		Pooled analysis		Sample 1		Sample 2		Pooled analysis	
	FR [a]	95 % CI	FR [a]	95 % CI	FR[b]	95 % CI	FR [a]	95 % CI	FR [a]	95 % CI	FR[b]	95 % CI
**PFOS**												
Q1	1.00		1.00		1.00		1.00		1.00		1.00	
Q2	1.00	0.77 – 1.31	0.78	0.66- 0.93	0.85	0.74 – 0.98	1.15	0.85 - 1.55	1.05	0.87 – 1.26	1.10	0.94 – 1.28
Q3	0.95	0.72 – 1.26	0.71	0.60 – 0.85	0.78	0.68 – 0.91	1.26	0.94 – 1.70	1.01	0.83 – 1.22	1.09	0.93 – 1.28
Q4	0.91	0.69 – 1.19	0.73	0.61 – 0.86	0.78	0.68 – 0.91	1.24	0.93 – 1.67	1.05	0.87 – 1.27	1.13	0.96 – 1.32
Log	0.87	0.69 – 1.09	0.71	0.60 – 0.85	0.77	0.67 – 0.86	1.26	0.99 – 1.59	1.00	0.84 – 1.20	1.10	0.96 – 1.27
**PFOA**												
Q1	1.00		1.00		1.00		1.00		1.00		1.00	
Q2	0.96	0.74 – 1.25	0.76	0.64 – 0.90	0.81	0.70 – 0.94	0.94	0.70 – 1.25	0.99	0.82 – 1.20	0.98	0.83 – 1.15
Q3	0.91	0.70 – 1.19	0.76	0.64 – 0.91	0.81	0.70 – 0.93	0.97	0.73 – 1.31	1.17	0.97 – 1.41	1.11	0.95 – 1.30
Q4	0.87	0.65 – 1.17	0.66	0.55 – 0.80	0.72	0.62 – 0.84	1.00	0.74 – 1.37	1.00	0.81 – 1.23	1.02	0.86 – 1.21
Log	0.86	0.68 – 1.08	0.68	0.59 – 0.79	0.73	0.65 – 0.83	1.02	0.79 – 1.32	1.00	0.85 – 1.19	1.02	0.89 – 1.18

[a]Adjusted for age, socio-economic status, body mass index, and parity. [b] Additionally adjusted for sample.

Abbreviations: Perfluorooctane sulfonate (PFOS), perfluorooctanoate (PFOA), quartile (Q), log-transformed continuous exposure (Log), time to pregnancy (TTP), fecundability ratio (FR), 95 % confidence interval (95 % CI).

Additional Table 5. Fecundability ratios according to plasma PFOS and PFOA without adjustment for parity

	Sample 1			Sample 2			Pooled analysis		
	Crude FR	Adjusted[a] FR	95 % CI	Crude FR	Adjusted[a] FR	95 % CI	Crude FR	Adjusted[b] FR	95 % CI
**PFOS**									
Q1	1.00	1.00		1.00	1.00		1.00	1.00	
Q2	1.06	1.06	0.80 – 1.42	0.79	0.77	0.64 – 0.92	0.85	0.85	0.73 – 0.99
Q3	0.99	0.98	0.73 – 1.32	0.80	0.73	0.60 – 0.88	0.84	0.80	0.69 – 0.94
Q4	0.91	0.93	0.69 – 1.25	0.75	0.70	0.58 – 0.84	0.79	0.77	0.66 – 0.90
Log	0.94	0.94	0.74 – 1.21	0.74	0.69	0.57 – 0.83	0.79	0.78	0.67 – 0.90
**PFOA**									
Q1	1.00	1.00		1.00	1.00		1.00	1.00	
Q2	0.91	0.86	0.64 – 1.16	0.75	0.71	0.59 – 0.85	0.79	0.75	0.64 – 0.87
Q3	0.90	0.88	0.66 – 1.17	0.80	0.71	0.60 – 0.85	0.82	0.76	0.65 – 0.88
Q4	0.74	0.71	0.52 – 0.96	0.69	0.59	0.49 – 0.71	0.70	0.63	0.53 – 0.73
Log	0.75	0.74	0.58 – 0.95	0.69	0.60	0.52 – 0.70	0.70	0.64	0.57 – 0.73

[a] Adjusted for age, socio-economic status, and body mass index.

[b] Adjusted for age, socio-economic status, body mass index, and sample.

Abbreviations: Perfluorooctane sulfonate (PFOS), perfluorooctanoate (PFOA), quartile (Q), log-transformed continuous exposure levels (Log), fecundability ratio (FR), 95 % confidence interval (95 % CI).

Additional Table 6. Fecundability ratios for PFOS and PFOA in Sample 1, unrestricted and restricted to those completing all four interviews.

	**Sample 1 unrestricted**		**Sample 1 restricted**	
	FR	95 % CI	FR	95 % CI
**PFOS**				
Q1	1.00		1.00	
Q2	1.08	0.81 - 1.44	1.09	0.73 – 1.65
Q3	0.99	0.73 - 1.34	0.96	0.66 – 1.40
Q4	0.99	0.74 - 1.33	0.95	0.68 – 1.45
Log	0.96	0.75 – 1.24	0.94	0.68 – 1.32
**PFOA**				
Q1	1.00		1.00	
Q2	0.92	0.69 - 1.22	0.91	0.61 – 1.35
Q3	0.94	0.71 - 1.26	1.10	0.75 – 1.63
Q4	0.86	0.63 - 1.19	0.91	0.58 – 1.43
Log	0.89	0.68 – 1.15	0.89	0.62 – 1.26

[a]Adjusted for age, socio-economic status, body mass index, and parity.

Abbreviations: Perfluorooctane sulfonate (PFOS), perfluorooctanoate (PFOA), quartile (Q), log-transformed continuous exposure (Log), time to pregnancy (TTP), fecundability ratio (FR), 95 % confidence interval (95 % CI).

References

Textor J, Hardt J, Knüppel S. DAGitty: a graphical tool for analyzing causal diagrams. *Epidemiol Camb Mass* 2011;**22**:745.
